# North Carolina pharmacists’ willingness to sell fentanyl test strips: a survey study

**DOI:** 10.1186/s12954-023-00739-4

**Published:** 2023-01-24

**Authors:** Grace T. Marley, Bayla Ostrach, Delesha Carpenter

**Affiliations:** 1 School of Pharmacy, UNC Eshelman, 201 Pharmacy Lane, CB 7355, Chapel Hill, NC 27599-7355 USA; 2 School of Medicine, Fruit of Labor Action Research and Technical Assistance, Boston University, LLC 85 E. Newton St., Boston, MA 02118 USA; 3 UNC Eshelman School of Pharmacy, 220 Campus Drive CPO 2125, Asheville, NC 28804 USA

**Keywords:** Fentanyl, Drug-checking, Pharmacist, Harm reduction

## Abstract

**Background:**

Although fentanyl test strips (FTS) can accurately determine the presence of fentanyl in unregulated substances, access to FTS remains limited. This study aimed to examine North Carolina community pharmacists’ attitudes and willingness to engage in various behaviors related to FTS sales and distribution.

**Methods:**

A convenience sample of community pharmacists completed an online survey that assessed: (1) comfort initiating an FTS conversation; (2) willingness to sell FTS, distribute FTS instructions, counsel on FTS, refer patients to harm reduction organizations, and advertise FTS; (3) perceived barriers and benefits of selling FTS; and (4) interest in FTS training. Data were collected from March to May 2022. Descriptive statistics were calculated.

**Results:**

Of the 592 pharmacists who participated, most were somewhat or very willing to refer patients to harm reduction organizations for FTS (514, 86.9%), counsel on FTS (485, 81.9%), distribute FTS instructions (475, 80.2%), sell FTS (470, 79.3%), and advertise FTS for sale (372, 62.9%). The most commonly reported benefits of selling FTS were reducing overdose deaths in the community (*n* = 482, 81.4%) and participating in community harm reduction efforts (*n* = 455; 76.9%). Barriers commonly reported to selling FTS were: not knowing where to order FTS (*n* = 295, 49.8%) and discomfort initiating a conversation about FTS (*n* = 266, 44.9%). Most respondents (88.3%) were interested in FTS training.

**Conclusion:**

North Carolina community pharmacists are willing to engage in various behaviors related to FTS sales and distribution. Most pharmacists were interested in receiving FTS training, which should be created to address pharmacist-reported barriers to FTS sales. Pharmacist distribution of FTS could increase access to FTS at the community level and has the potential to change drug use behavior and reduce overdose deaths.

**Supplementary Information:**

The online version contains supplementary material available at 10.1186/s12954-023-00739-4.

## Background

According to provisional data from the Centers for Disease Control and Prevention (CDC), there were an estimated 107,622 drug overdose deaths in the USA in 2021, with synthetic opioids, including non-prescribed fentanyl, present in 66% of those deaths [1, 2]. Fentanyl is 50–100 times more potent than heroin, with a rapid onset of action and relatively short duration of effect [3]. In some cases, people who use drugs (PWUD) unknowingly consume fentanyl or are unaware of the ratio of fentanyl to other substances in what they consume, which puts them at greater risk of overdose. In North Carolina (NC), fentanyl-positive overdose deaths increased by 212% from 1490 deaths in 2019 to 3163 deaths in 2021 [4].

In April 2022, the Biden-Harris administration released a National Drug Control Strategy that calls for expanded access to evidence-based harm reduction interventions including fentanyl test strips (FTS), as part of a multipronged approach to reduce overdose deaths [2]. FTS are a simple, inexpensive drug-checking device that can detect the presence of fentanyl and some fentanyl analogs in a substance prior to consumption [10]. Existing research on FTS is limited but growing, with early studies documenting both the need for FTS and the potential benefit of FTS to reduce overdose risk among PWUD [5, 6]. FTS may encourage safer drug use behaviors among PWUD, such as using less of a substance or not using alone [6–9]. A study in Rhode Island among young (18–35 years) self-reported PWUD showed high levels (> 90%) of willingness to use FTS [5].

Currently, access to FTS is limited [10]. FTS are commonly distributed by harm reduction organizations, with research indicating over 77% of syringe services programs offering them in 2019 [11]. However, these programs are often limited geographically, being largely concentrated in urban areas with less coverage in the South and Appalachia [12]. A systematic legal review found that it is clearly legal to distribute drug-checking equipment in 19 states, and in 14 states where distribution is not clearly legal, it is legal when that equipment is obtained from an SSP [13]. According to qualitative interviews with PWUD, there is a need to increase access to and awareness of FTS, with some users suggesting pharmacies as good locations to receive FTS [10].

Community pharmacists are the most accessible healthcare providers for the general public, especially in rural and underserved communities [14]. Pharmacists play a vital role in overdose risk reduction through opioid medication counseling and the dispensing of naloxone [15]. Although pharmacists are actively engaged with overdose reduction strategies, no published research reports on pharmacist awareness of and willingness to sell FTS [16].

The objective of this study was to determine NC community pharmacists’ attitudes toward willingness to engage in various FTS behaviors and to document pharmacist perceptions of barriers to and benefits of selling FTS. Pharmacists’ interest in completing FTS training was also gauged. NC is an ideal state to conduct this study because in 2019, the General Assembly amended G.S. 90-113.22—under which the NC Board of Pharmacy (NCBOP) staff and the legal adviser to the NC Department of Health and Human Services agree that pharmacies may distribute testing equipment such as FTS [16].

## Methods

### Participants

A convenience sample was recruited from the 4644 currently practicing community pharmacists who had a valid email address on file with the NCBOP. These pharmacists were invited to participate in a 10-min, confidential, online survey. The survey instrument can be found in Additional file 1. Pharmacists were eligible to participate if they currently worked in a community pharmacy setting, defined as a pharmacy that dispenses medications to patients for use at their home (e.g., chain pharmacy, grocery store pharmacy, independent pharmacy, hospital outpatient pharmacy, etc.).

### Procedures

All eligible participants received an email describing the study and a link to the Qualtrics survey. The first page included a study fact sheet containing all elements of informed consent and a survey link. Approximately 2 weeks after the initial email, the first author sent a follow-up email to a random sample of at least three non-responding pharmacists in each county. In addition, this survey was advertised through the North Carolina Association of Pharmacists email listserv. If the county had fewer than three registered pharmacists, all pharmacists were contacted. This approach was chosen to increase geographical representativeness of the sample. Additionally, four reminder emails were sent to the full email list. The survey remained open between March 10, 2022, and May 5, 2022.

Because measures to assess pharmacists’ attitudes toward and willingness to engage in various FTS behaviors did not exist at the time this survey was distributed, new measures were developed to document pharmacist perceptions of barriers to and benefits of selling FTS. Two ‘select all that apply’ questions regarding perceived benefits and barriers of FTS at the pharmacy were created utilizing the previous harm reduction literature [5, 10, 17]. The technical functionality of the 23-item Qualtrics survey was beta-tested prior to distribution.

Pharmacists who voluntarily completed the survey could enter a separate drawing for one of 10 $100 Amazon gift cards. Contact information could not be linked to survey responses. The University of North Carolina at Chapel Hill Institutional Review Board reviewed this study and deemed it exempt from further review.

### Measures

Pharmacists answered seven multiple-choice demographic questions, including age, gender, years of practice, and practice setting. Pharmacist comfort with initiating a conversation about FTS was measured with one item on a 5-point Likert scale (1 = not at all comfortable; 5 = completely comfortable). Respondents' willingness to advertise, sell, counsel, and distribute FTS instructions, and willingness to refer patients to harm reduction organizations were evaluated with 5 items measured on a 5-point scale (1 = not at all willing, 2 = slightly willing, 3 = somewhat willing, 4 = very willing, 5 = already in practice at my location). Pharmacists were asked to select all that apply with up to seven perceived benefits of selling FTS including: *reduce overdose deaths in the community, participate in harm reduction efforts in my community, engage customers that may otherwise feel stigmatized, reduce harm for patients when unable to dispense opioids or buprenorphine, new source of revenue for the pharmacy, I do not believe there are any benefits to selling FTS; other*). Pharmacists indicated perceived barriers to selling FTS through a ‘select all that apply’ question, from a list of eight barriers: *unaware of where to order FTS, discomfort initiating a conversation about FTS, concern for legality, do not want to attract individuals with substance use disorder (SUD) to my pharmacy, lack of time to educate about FTS, identifying patients who would benefit from FTS, lack of interest in selling FTS, other*). One question assessed pharmacists' interest in completing an FTS training (with responses ranging from 1 = not at all interested to 4 = very interested).

### Data analysis

IBM SPSS Statistics (Version 26) was used to analyze the data. Descriptive statistics, including frequencies and means, were used to characterize the sample and variables of interest.

## Results

**Fig. 1:**
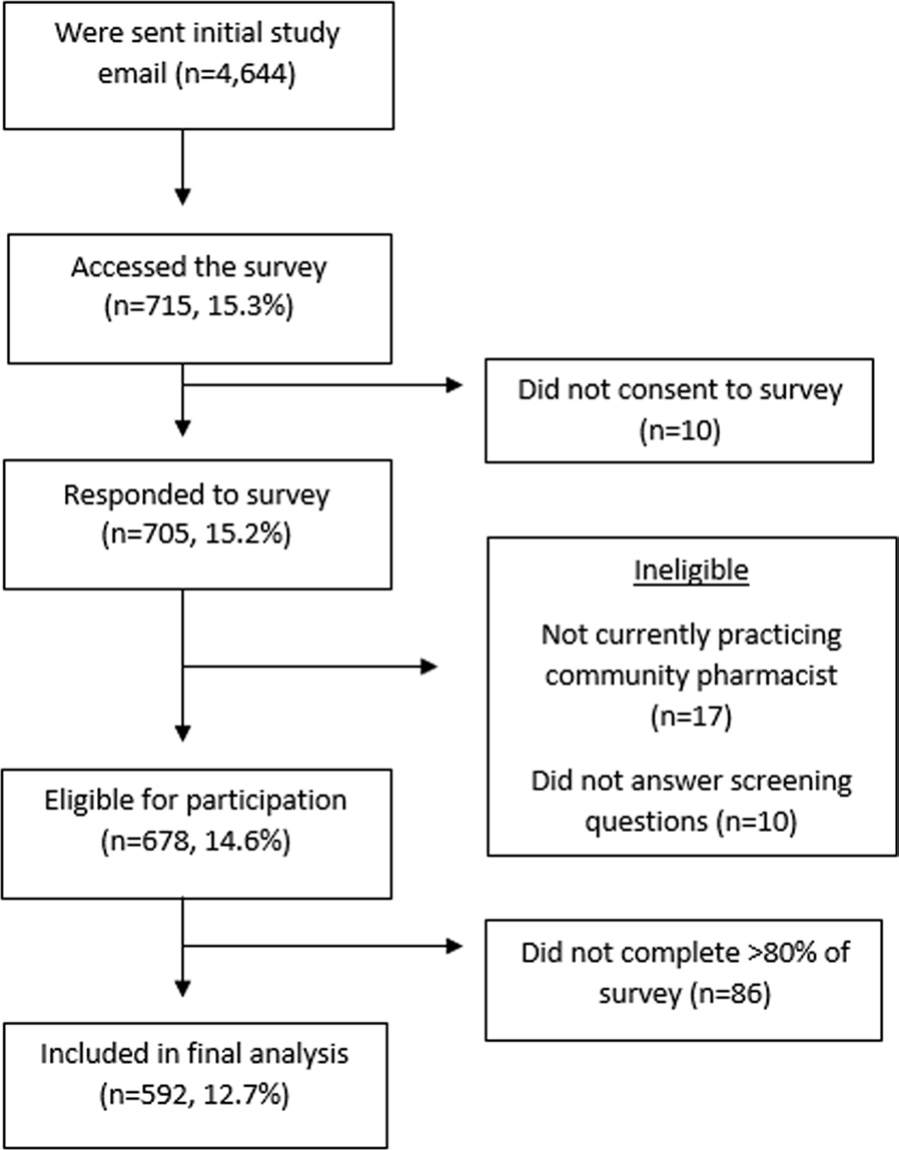
Recruitment flowchart for study sample

As noted in Fig. 1, 592 pharmacists were included in the final analysis, resulting in a survey completion rate of 12.7%. Demographic characteristics are presented in Table 1.

**Table 1:** Sample characteristics (*N* = 592)

Characteristic	*N* (592)	% (Of survey respondents)
Age		
18–29	89	15.1
30–39	187	31.6
40–54	211	35.7
55+	103	17.5
Gender^a^		
Female	383	64.7
Male	201	34
Non-binary or transgender	3	0.5
Prefer not to answer	4	0.7
Race/ethnicity		
Asian or Pacific Islander	25	4.2
Black or African-American	26	4.4
Native American or Alaska Native	5	0.8
White or Caucasian	509	86
Multiracial or Biracial	7	1.2
Length of time at current pharmacy		
0–3 years	239	40.4
4–11 years	216	36.5
≥ 12 years	135	22.8
Type of pharmacy practice		
Independent pharmacy	257	43.4
National chain	231	39
Grocery store chain	71	12
Other	19	3.2
Regional chain	10	1.7

^a^Gender was selected from female, male, non-binary or transgender, or prefer not to answer

### Pharmacist willingness to engage in FTS behaviors

The majority of pharmacists were somewhat or very willing to refer patients to harm reduction organizations for FTS (86.9%), counsel on FTS (81.9%), distribute FTS instructions (80.2%), sell FTS (79.3%), and advertise FTS for sale (62.9%) (Table 2). Pharmacists were less willing to advertise FTS for sale (62.9%). Only two pharmacies currently distributed FTS instructions and counseled on how to use FTS. Community partners later informed surveyors that the two pharmacies that engaged in FTS behaviors have ongoing partnerships with a local syringe service program, to offer these services. Pharmacists were overwhelmingly willing to refer patients to harm reduction organizations for FTS (86.2%), with four pharmacists (0.7%) noting they already referred patients to harm reduction organizations. No pharmacists noted that they advertised FTS for sale at their pharmacy.

**Table 2:** Pharmacist willingness to engage in various FTS behaviors (*N* = 592)

	Not at all willing	Slightly willing	Somewhat willing	Very willing	Already in practice at my location
	*n* (%)	*n* (%)	*n* (%)	*n* (%)	*n* (%)
Sell FTS	46 (7.8)	74 (12.5)	147 (24.8)	321 (54.2)	2 (0.3)
Distribute FTS instructions	41 (6.9)	75 (12.7)	167 (28.2)	306 (51.7)	2 (0.3)
Counsel on how to use FTS	33 (5.6)	73 (12.3)	146 (24.7)	337 (56.9)	2 (0.3)
Refer to harm reduction organizations for FTS	20 (3.4)	58 (9.8)	136 (23)	374 (63.2)	4 (0.7)
Advertise FTS for sale at pharmacy	103 (17.4)	116 (19.6)	149 (25.2)	223 (37.7)	–

### Comfort counseling on FTS

Regarding pharmacists’ comfort to initiate a conversation about FTS, 92 (15.5%) were not at all comfortable, 198 (33.4%) were slightly uncomfortable, 89 (15%) were neither comfortable nor uncomfortable, 133 (22.5%) were somewhat comfortable, and 78 (13.2%) were completely comfortable.

### Perceived benefits of selling FTS

The most common perceived benefits to selling FTS were: reducing overdose deaths (*n* = 482, 81.4%) and participating in harm reduction efforts (*n* = 455, 76.9%). Less endorsed benefits were: engaging customers who may otherwise feel stigmatized (*n* = 227, 38.3%), reducing harm for patients when unable to dispense opioids or buprenorphine (*n* = 222, 37.5%), and initiating a new source of revenue for the pharmacy (*n* = 112, 18.9%). Some pharmacists (*n* = 38, 6.4%) did not believe that there were any benefits to selling FTS.

### Perceived barriers to selling FTS

The most common perceived barriers to selling FTS were: not knowing where to order FTS (*n* = 295, 49.8%), lack of time to educate about FTS (*n* = 271; 45.8%), discomfort initiating an FTS conversation (*n* = 266, 44.9%), and not wanting to attract individuals with substance use disorders to the pharmacy (*n* = 249, 42.1%). Pharmacists also were concerned about the legality of selling FTS (*n* = 108, 18.2%).

### Interest in FTS training

Most pharmacists (*n* = 522, 88.3%) expressed some interest in receiving training on FTS, with 20.9% (*n* = 124) very interested, 34.5% (*n* = 204) somewhat interested, and 32.8% (*n* = 194) slightly interested. Approximately 12% (*n* = 69) were not interested in FTS training.

## Discussion

This is the first study to report on the willingness of community pharmacists to engage in various FTS behaviors. Many NC community pharmacists in this sample were somewhat or very willing to sell FTS as well as counsel on FTS, which could greatly increase FTS access, especially in communities without harm reduction organizations. Despite this high degree of willingness, only two pharmacies were currently providing FTS. This could be due to logistical and attitudinal barriers.

FTS are a relatively new harm reduction tool, and as such there is limited awareness of FTS among both PWUD and pharmacists. Among a sample of rural Appalachian PWUD, most were unaware of FTS, but once they learned about them, they perceived FTS utilization as a viable overdose harm reduction tool ^9^. Similarly, pharmacists in this sample reported not knowing where to order FTS. Although pharmacists were unaware of where to obtain FTS, the majority perceived that FTS could reduce overdose deaths in their communities.

Pharmacists are already engaged in harm reduction behaviors at their pharmacies. A survey of Rhode Island practitioners found that pharmacists felt it was their role to provide equipment and advice to injection drug users to prevent the spread of infections [18]. Additionally, a survey of North Carolina pharmacists indicated that pharmacists already engage in harm reduction strategies like selling non-prescription syringes, dispense naloxone, and occasionally test for HIV and HCV [19]. Overwhelmingly, pharmacists in this sample are willing to add FTS sales to this list of harm reduction strategies, with two pharmacies already partnering with a local syringe service program to offer FTS. Although pharmacists are interested in selling FTS, logistical barriers such as store-level policies and lack of time to educate pharmacists may limit the sale of FTS in community pharmacies [20]. Community pharmacies are easily accessible and could offer a solution to improve access to syringe service programs and reduce risk behaviors among people who use drugs, if given the proper support by leadership [21]. Future work should explore the FTS opinions of pharmacy managers.

Although studies have not examined the effect of training on pharmacy FTS sales, parallels may be drawn with pharmacy-based naloxone distribution. Research on public health partnerships with community pharmacies has led to increased naloxone protocols and dispensing [21]. Training on naloxone counseling for community pharmacists resulted in a reduction in stigmatizing attitudes and an increase in pharmacist-reported confidence to appropriately identify, discuss, and dispense naloxone [22]. Pharmacist trainings should be created regarding FTS counseling similar to trainings regarding naloxone counseling in an effort to increase pharmacist comfort and confidence in FTS distribution. Through the implementation of harm reduction activities like selling FTS, naloxone distribution, and non-prescription syringe sales at community pharmacies, a social norm is created in that pharmacies are locations of harm reduction and are avenues in which further harm reduction efforts can take place.

Stigma has been identified as a contributing factor to reduced access to harm reduction programs [23]. Almost half of the pharmacists in this survey indicated that they did not want to attract customers with SUD to their pharmacy. Of note, the implementation of harm reduction programs in community pharmacies did not result in reduced clientele or a significant increase in criminal activity in the store according to quantitative surveys of pharmacies in SF and LA counties [24, 25]. In order to overcome the stigma associated with not wanting to attract customers with SUD to the pharmacy and increase patient access to FTS, training should address how to engage with patients in a non-stigmatizing way. Many North Carolina community pharmacists noted that they were interested in receiving training regarding FTS. Training should be developed to address commonly reported barriers to selling FTS, such as information on where and how to order FTS, and how to best counsel patients on FTS in a manner that fits into workflow. Training should also emphasize the benefits of FTS, especially reducing overdose deaths. Because many of these desired training topics were logistical in nature, the creation of FTS training that provides information on important basic topics around ordering, selling, and counseling on FTS can assist pharmacists in implementing this important harm reduction strategy in their pharmacies. The creation of training for pharmacists regarding FTS is desired from NC community pharmacists and can assist the implementation of this harm reduction strategy by addressing the reported barriers by pharmacists.

### Limitations

This study possesses several limitations. First, the completion rate was low (12.75%) but higher than other surveys that have used this same sample [17, 26]. The low completion rate could result in selection bias whereby only pharmacists who were more interested in FTS completed the survey, which could affect generalizability. Results are not generalizable to all other states, such as Texas, where the sales and distribution of FTS is illegal [27]. Further work should investigate whether PWUD would be willing to purchase FTS from pharmacies. Also, as there were no existing measures to assess pharmacists FTS attitudes and behaviors, we adapted items from other harm reduction scales when possible and created new items, when necessary, which means that the validity of these measures has not been established. Future work should explore construct validity of these measures.

## Conclusion

Many NC community pharmacists are willing to sell FTS and engage in other FTS behaviors. Pharmacists in this survey were willing to partner with and refer patients to harm reduction organizations and could be an incredible resource to reduce harm. Pharmacists perceived more barriers than benefits to selling FTS, indicating that FTS training should address ways to overcome barriers, including where to order FTS and how to initiate FTS conversations. It is important for FTS training to address stigmatizing attitudes and emphasize the benefits of FTS. Access to FTS could be greatly increased in many communities through pharmacy sales.

## Supplementary Information

**Additional file 1.** The 23- item Qualtrix survey instrument utilized to gauge NC Community pharmacists' willingness to engage in various FTS behaviors, and demographic data.

## Data Availability

The datasets used and/or analyzed during the current study are available from Dr. Delesha Carpenter upon reasonable request.

## Abbreviations

CDC: Centers for Disease Control and Prevention
FTS: Fentanyl test strips
NC: North Carolina
NCBOP: North Carolina Board of Pharmacy
PWUD: People who use drugs
SSP: Syringe service program
SUD: Substance use disorder
