# Supplementary material for: North Carolina pharmacists’ willingness to sell fentanyl test strips: a survey study
Source: Harm Reduct J. 2023 Jan 24;20:10. doi: 10.1186/s12954-023-00739-4 (PMC9875410; doi:10.1186/s12954-023-00739-4)
Supplement: Supplementary file 1 — Additional file 1. The 23- item Qualtrix survey instrument utilized to gauge NC Community pharmacists' willingness to engage in various FTS behaviors, and demographic data. [file 12954_2023_739_MOESM1_ESM.docx]

APPENDIX:

Fentanyl Test Strip Awareness Survey

How comfortable would you be initiating a conversation about fentanyl test strips with customers who you think would benefit from them?

- Not at all comfortable
- Slightly uncomfortable
- Neither comfortable nor uncomfortable
- Somewhat comfortable
- Completely comfortable

Q7 How willing would you be to do the following activities with customers who you think would benefit from them?

|  | Not at all willing (1) | Slightly willing (2) | Somewhat willing (3) | Very willing (4) | Already in practice at my location (5) |
| --- | --- | --- | --- | --- | --- |
| Distribute fentanyl test strip instructions (1) |  |  |  |  |  |
| Counsel on how to use fentanyl test strips (2) |  |  |  |  |  |
| Refer patients to harm reduction organizations for fentanyl test strips (3) |  |  |  |  |  |
| Sell fentanyl test strips (4) |  |  |  |  |  |
| Advertise fentanyl test strips for sale at pharmacy (5) |  |  |  |  |  |

Q8 What do you perceive to be the benefits of selling fentanyl test strips at your pharmacy? *Check all that apply.*

- Reduce overdose deaths in community
- Participate in harm reduction efforts in my community
- Engage customers that may otherwise feel stigmatized
- Reduce harm for patients when unable to dispense opioids or buprenorphine
- New source of revenue for the pharmacy
- Other (please specify) __________________________________________________
- I do not believe there are any benefits to selling fentanyl test strips

Q9 What might make it harder for you to sell fentanyl test strips? *Check all that apply.*

- I don't know where to order fentanyl test strips
- Discomfort initiating a conversation about fentanyl test strips
- Concern about legality
- Do not want to attract individuals with substance use disorders to my pharmacy
- Lack of time to educate about fentanyl test strips
- Identifying patients who would benefit from fentanyl test strips
- Lack of interest in selling fentanyl test strips
- Other (please specify) __________________________________________________

Q10 How interested would you be in completing a training on fentanyl test strips?

- Not at all interested
- Slightly interested
- Somewhat interested
- Very interested

Q11 What would be the best way to deliver this training?

- Webinar with live CE credit
- On-demand online CE program with correspondence CE credit
- In-person session at a conference or professional meeting with CE credit
- Other (please specify) __________________________________________________

Q12 Would you be interested in training on any of the following other topics?

|  | Yes (1) | No (2) |
| --- | --- | --- |
| Increasing buprenorphine order sizes from wholesaler (1) |  |  |
| How to enter into a dedicated dispensing agreement with a prescriber (2) |  |  |
| How to create a welcoming environment for buprenorphine patients (3) |  |  |

**The last set of questions asks about demographic characteristics and characteristics of your pharmacy.**

Q13 What is your age?

- 18-24
- 25-29
- 30-34
- 35-39
- 40-44
- 45-54
- 55-64
- 65-74
- 75 and older

Q14 What is your gender?

- Female
- Male
- Non-binary or transgender
- Prefer not to answer

Q15 Which of the following best describes your race/ethnicity?

- Asian or Pacific Islander
- Black or African American
- Hispanic or Latino
- Native American or Alaska Native
- White or Caucasian
- Multiracial or Biracial
- Other (Please specify) __________________________________________________
- Prefer not to answer

Q16 How long have you worked in pharmacy practice?

- Less than 1 year
- 1-3 years
- 4-7 years
- 8-11 years
- 12-15 years
- Greater than 16 years

Q17 How long have you worked at your current pharmacy?

- Less than 1 year
- 1-3 years
- 4-7 years
- 8-11 years
- 12-15 years
- Greater than 16 years

For the next set of questions, if you work in more than one pharmacy, please **think about the pharmacy where you work most.**

Q18 What type of pharmacy do you work at?

- Independent pharmacy
- Grocery store chain
- Regional chain
- National chain
- Other (Please specify) __________________________________________________

Q19 What is the zip code at your pharmacy? ________________________________

Q20 What harm reduction services does your pharmacy currently offer? *Check all that apply.*

- Naloxone dispensing
- Selling non-prescription syringes
- HCV screening
- HIV rapid testing
- Biohazard (syringe disposal) container on site accessible to patients
- None of the above
- Other (please specify) ___________________________________________
